# Antithrombotics prescription and adherence among stroke survivors: A systematic review and meta‐analysis

**DOI:** 10.1002/brb3.2752

**Published:** 2022-09-06

**Authors:** Min Yang, Hang Cheng, Xia Wang, Menglu Ouyang, Sultana Shajahan, Cheryl Carcel, Craig Anderson, Espen Saxhaug Kristoffersen, Yapeng Lin, Else Charlotte Sandset, Xiaoyun Wang, Jie Yang

**Affiliations:** ^1^ Department of Neurology The First Affiliated Hospital of Chengdu Medical College Chengdu China; ^2^ The George Institute for Global Health, Faculty of Medicine University of New South Wales NSW Australia; ^3^ Department of Neurology, Royal Prince Alfred Hospital The University of Sydney NSW Australia; ^4^ The George Institute China at Peking University Health Science Centre Beijing PR China; ^5^ Department of Neurology Akershus University Hospital Lørenskog Norway; ^6^ Department of General Practice, Helsam University of Oslo Oslo Norway; ^7^ International Clinical Research Center Chengdu Medical College Chengdu China; ^8^ Stroke Unit, Department of Neurology Oslo University Hospital Oslo Norway; ^9^ The Norwegian Air Ambulance Foundation Oslo Norway; ^10^ Department of Neurology Affiliated Drum Tower Hospital of Nanjing University Medical School Nanjing China; ^11^ Department of Neurology Sichuan Provincial People's Hospital University of Electronic Science and Technology of China Chengdu China

**Keywords:** anticoagulant, antiplatelet, antithrombotic, secondary prevention, stroke, systematic review

## Abstract

**Objectives:**

We aimed to investigate the prescription of antithrombotic drugs (including anticoagulants and antiplatelets) and medication adherence after stroke.

**Methods:**

We performed a systematic literature search across MEDLINE and Embase, from January 1, 2015, to February 17, 2022, to identify studies reporting antithrombotic medications (anticoagulants and antiplatelets) post stroke. Two people independently identified reports to include, extracted data, and assessed the quality of included studies according to the Newcastle–Ottawa scale. Where possible, data were pooled using random‐effects meta‐analysis.

**Results:**

We included 453,625 stroke patients from 46 studies. The pooled proportion of prescribed antiplatelets and anticoagulants among patients with atrial fibrillation (AF) was 62% (95% CI: 57%–68%), and 68% (95% CI: 58%–79%), respectively. The pooled proportion of patients who were treated according to the recommendation of guidelines of antithrombotic medications from four studies was 67% (95% CI: 41%–93%). It was reported that 11% (95% CI: 2%–19%) of patients did not receive antithrombotic medications. Good adherence to antiplatelet, anticoagulant, and antithrombotic medications was 78% (95% CI: 67%–89%), 71% (95% CI: 57%–84%), and 73% (95% CI: 59%–86%), respectively.

**Conclusion:**

In conclusion, we found that less than 70% of patients were prescribed and treated according to the recommended guidelines of antithrombotic medications, and good adherence to antithrombotic medications is only 73%. Prescription rate and good adherence to antithrombotic medications still need to be improved among stroke survivors.

## Graphical Abstract

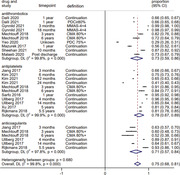

## 1 INTRODUCTION

Recurrent strokes account for approximately 20% of all strokes (Benjamin et al., 2018). The cause of stroke recurrence includes nonadherence to antithrombotic treatment (Broderick et al., 2011). Antithrombotic agents (anticoagulants and antiplatelets) are among important factors to prevent short‐ and long‐term recurrence of ischemic stroke (Del Brutto et al., 2019). Patients with stroke with high medication adherence have lower incidence of adverse outcomes compared to those with low medication adherence (Kim et al., 2017; Perreault et al., 2012; Rijkmans et al., 2018). Despite this, the secondary prevention measures after stroke have shown significant gaps in specialist care, monitoring, and treatment programs (Broderick et al., 2011; Webb et al., 2019; Weimar et al., 2013). The European Stroke Action Plan (ESAP) for the years 2018–2030 outlined targets for the development of stroke care, one of which is secondary prevention and organized follow‐up (Norrving et al., 2018).

To summarize the prescription rate and patient medication adherence of antithrombotics after stroke, we conducted this systematic review and meta‐analysis synthesizing the evidence on the optimal antithrombotic treatment and adherence according to guidelines for the secondary prevention of stroke.

## 2 MATERIALS AND METHODS

The systematic review was reported following Meta‐analysis Of Observational Studies in Epidemiology (MOOSE) guidelines (Stroup et al., 2000). We reviewed only previously published data, and ethics committee approval and all subjects informed consent were not required.

### 2.1 Search strategy

A comprehensive search strategy (the Appendix), which was developed in consultation with a university librarian, neurologists, and epidemiologists, was used to address the unique features and indexing of each of the two electronic databases (Medline and Embase) that were searched from January 1, 2015, to February 17, 2022. As well as searching for original studies, the reference lists of any relevant reviews appearing in their reports were examined.

### 2.2 Selection criteria

Any studies reporting antithrombotic medications (anticoagulants and antiplatelets) after stroke (ischemic or hemorrhagic) or transient ischemic attack (TIA) were included. Patients aged 18 years and over, of any race with a clinical or imaging (computed tomography [CT] or magnetic resonance imaging [MRI]) diagnosis of stroke, were included. There were no language restrictions.

### 2.3 Data extraction and quality assessment

MY and HC independently screened the titles and abstracts, excluded irrelevant references, and reviewed abstracts of potential relevance to identify reports for review in full text. MY and HC extracted data independently from the included studies. MO and SS assessed the quality of included studies according to the Newcastle–Ottawa scale (NOS) (Stang, 2010). Any disagreements were resolved by a third author (XW or JY).

### 2.4 Outcomes

The main outcomes were the proportion of patients prescribed and using (adherence) antithrombotic medication after stroke. Medication adherence refers to the extent to which patients act in accordance with the prescribed interval and dose of the medication regimen. Medication persistence was defined as the duration of time from initiation to discontinuation of therapy (Cramer et al., 2008). Good adherence to the medications was defined by continuation of medications (Ullberg et al., 2017) or prescription refill, for example, Continuous Measure of Medication Acquisition (CMA) (Hess et al., 2006), the proportion of days covered (PDC) (Yeo et al., 2020), or the 4‐item Morisky Medication Adherence Scale (MMAS‐4) (Morisky et al., 1986).

### 2.5 Statistical analysis

The data were pooled using random‐effects models where data were available. An *I* ^2^ statistic was considered to reflect low likelihood (0%−25%), moderate likelihood (26%−75%), and high likelihood (76%−100%) of differences beyond chance, as was a *P* value of less than or equal to 0.05 for heterogeneity (Rothman et al., 2008). Statistical analysis was performed with Stata, version 16.

## 3 RESULTS

Of 54,407 references identified through the databases, 109 remained after screening titles and abstracts for relevance (Figure S1). Forty‐six studies (453,625 patients) that satisfied the eligibility criteria were included in the review (Table 1). Of the 46 studies, 31 studies reported prescription of antiplatelets, and 11 reported anticoagulation among patients with atrial fibrillation (AF). Two studies were defined as low quality (scores < 5) on the NOS (Table 2).

**TABLE 1:** Included studies

Author	Country	Study design	Subtype	Number of subjects	Age (mean, SD)	Sex (female, %)
Bergstrom 2017 (Bergstrom et al., 2017)	Sweden	Population‐based study/national registry	Ischemic stroke	196765	76 (11.4)	50
Mechtouff 2018 (Mechtouff et al., 2018)	France	Single‐center hospital‐based study	Ischemic stroke or tia	373	<60 (24.9%)	43
Faure 2020 (Faure et al., 2020)	Canada	Population‐based study/national registry	Ischemic stroke	5587	<65 (17.3%)	50
Jithin 2016 (Jithin et al., 2016)	India	Single‐center hospital‐based study	Ischemic stroke	295	<60 (42.0%)	39
Eriksson 2017 (Eriksson, 2017)	Sweden	Single‐center hospital‐based study	Stroke	549	70	48
Rijkmans 2018 (Rijkmans et al., 2018)	The Netherlands	Single‐center hospital‐based study	Ischemic stroke	286	70	48
Desmaele 2016 (Desmaele et al., 2016)	International	Multi‐center hospital‐based study	Stroke	247	68.6 (60.0‐75.4)	47
Zhang 2017 (Zhang et al., 2017)	China	Multi‐center hospital‐based study	Ischemic stroke & AF	1014	70.3 (10.8)	54
Lim 2015 (Lim et al., 2015)	Korea	Multi‐center hospital‐based study	Tia	500	64.4 (11.8)	42
Park 2017 (Park et al., 2017)	Korea	Multi‐center hospital‐based study	Ischemic stroke or tia	9506	65.9 (12.7)	39
Ullberg 2017 (Ullberg et al., 2017)	Sweden	Population‐based study/national registry	Ischemic stroke	5602	73	47
Sarfo 2016 (Sarfo et al., 2017)	Ghana	Single‐center hospital‐based study	Stroke	418	60	50
Sluggett 2015 (Sluggett et al., 2015)	Australia	Population‐based study/national registry	Ischemic stroke or tia	1541	85	51
Jiang 2017 (Jiang et al., 2017)	China	Population‐based study/national registry	Ischemic stroke or tia	18344	64 (56‐73)	36
Brewer 2015 (Brewer et al., 2015)	United Kingdom	Multi‐center hospital‐based study	Ischemic stroke	302	> = 65 (66%)	43
Haeusler 2015 (Haeusler et al., 2015)	Germany	Population‐based study/national registry	Ischemic stroke or tia & af	896	71.3 (9.6)	43
Yeo 2020 (Yeo et al., 2020)	Singapore	Population‐based study/national registry	Ischemic stroke	1215	65.3 (13.4)	38
Mazurek 2017 (Mazurek et al., 2016)	United Kingdom	Population‐based study/national registry	Stroke & AF	428	79.6 (9.6)	45
Abdo 2019 (Abdo et al., 2019)	Lebanon	Multi‐center hospital‐based study	Ischemic stroke or TIA	173	69.8 (12.7)	40
Magwood 2017 (Magwood et al., 2017)	United States	Population‐based study/national registry	Stroke	125	39.6 (7.7)	54
Akijian 2017 (Akijian et al., 2017)	United Kingdom	Population‐based study/national registry	TIA	172	71 (12.2)	51
Akijian 2017	United Kingdom	Population‐based study/national registry	Ischemic stroke	412	71.4 (13.4)	49
Sauer 2015 (Sauer et al., 2015)	Germany	Single‐center hospital‐based study	Ischemic stroke & AF	284	78.1 (9.5)	51
Xian 2015 (Xian et al., 2015)	United States	Population‐based study/national registry	Ischemic stroke & AF	12552	80.5 (7.6)	60
Shah 2016 (Shah et al., 2016)	Canada	Multi‐center hospital‐based study	Ischemic stroke or TIA & AF	5781	–	46
Guidoux 2019 (Guidoux et al., 2019)	France	Multi‐center hospital‐based study	Stroke & AF	400	78.7 (11.0)	52
Xu 2017 (Xu et al., 2016)	China	Single‐center hospital‐based study	Ischemic stroke	878	63.2 (13.1)	35
Jurjans 2019 (Jurjans et al., 2019)	Latvia	Single‐center hospital‐based study	Ischemic stroke & AF	682	80 (75‐85)	69
Saade 2021 (Saade et al., 2021)	Lebanon	Multi‐center hospital‐based study	Ischemic stroke	100	74.0 (10)	43
Gynnild 2021 (Gynnild et al., 2021)	Norway	Multi‐center hospital‐based study	Ischemic stroke	664	72.9 (11.5)	43
Dalli 2020 (Dalli et al., 2021)	Australia	Population‐based study/national registry	Stroke or TIA	9817	74.2 (63.3, 82.5)	45
Yeo 2020 (Yeo et al., 2020)	Singapore	Population‐based study/national registry	Ischemic stroke	3469	–	44
Shankari 2020 (Shankari et al., 2020)	Singapore	Single‐center hospital‐based study	Ischemic stroke or TIA	199	62.9 (11.9)	36
Malaeb 2020 (Malaeb et al., 2020)	Lebanon	Multi‐center hospital‐based study	Ischemic stroke	204	65.4 (11.9)	33.3
MacDonald 2020 (MacDonald et al., 2020)	United States	Multi‐center hospital‐based study	Stroke	107	56.0 (11.2)	42.1
Gronemann 2020 (Gronemann et al., 2020)	Germany	Population‐based study/national registry	Ischemic stroke & AF	1512	76.7 (9.6)	53.3
Flach 2020 (Flach et al., 2020)	United Kingdom	Population‐based study/national registry	Stroke	6052	<65 (34%)	49
Chang 2020 (Chang et al., 2020)	United States	Population‐based study/national registry	Stroke & AF	64228	84 (78‐89)	63
Abanto 2020 (Abanto et al., 2020)	Peru	Population‐based study/national registry	Stroke	150	66.3 (12.6)	38
Chen 2019 (Chen et al., 2019)	Canada	Multi‐center hospital‐based study	Ischemic stroke or TIA	408	68 (13)	47.5
Chen 2019	Canada	Multi‐center hospital‐based study	Ischemic stroke or TIA	392	70 (11)	43.1
Dalli 2021 (Dalli et al., 2021)	Australia	Multi‐center hospital‐based study	Stroke or tia	8363	≥75 (44%)	44
Kim 2021 (Kim et al., 2021)	South Korea	Population‐based study/national registry	Ischemic stroke	4621	66.4 (12.3)	43.8
Kothagundla 2021 (Kothagundla et al., 2021)	India	Single‐center hospital‐based study	Stroke	150	60 (1)	37
Preinreich 2021 (Preinreich et al., 2021)	Austria	Population‐based study/national registry	Stroke	76354	–	–
Rodríguez‐Bernal 2021 (Rodríguez‐Bernal et al., 2021)	Spain	Population‐based study/national registry	Ischemic stroke or TIA & AF	10986	78.8 (9.3)	53.3
Sheehan 2021 (Sheehan et al., 2021)	United States	Population‐based study/national registry	Ischemic stroke	172	75.0 (7.3)	–
Tiili 2021 (Tiili et al., 2021)	Finland	Population‐based study/national registry	Ischemic stroke & AF	396	75.0 (70−80)	43

AF: atrial fibrillation; IS: ischemic stroke; TIA: transient ischemic stroke.

**TABLE 2:** Quality assessment for the included studies

Study	Study type	Selection_1	Selection_2	Selection_3	Selection_4	Comparability	Outcome_1	Outcome_2	Outcome_3	Total scale
Abdo, 2019	Cohort	1	1	0	1	0	0	0	1	4
Akijian, 2017	Cohort	1	1	1	1	2	1	0	1	8
Bergstrom, 2017	Cohort	1	1	1	1	2	1	1	1	9
Brewer, 2015	Cohort	1	1	1	1	0	1	1	0	6
Chen, 2019	Cohort	1	1	1	1	2	1	1	0	8
Dalli, 2021	Cohort	1	1	1	1	2	1	1	0	8
Desmaele, 2016	Cohort	1	1	1	1	0	0	1	0	5
Eriksson, 2017	Cohort	0	1	1	1	0	1	1	1	6
Kim, 2021	Cohort	1	1	1	1	2	1	1	1	9
Faure, 2020	Cohort	1	1	1	1	2	1	1	1	9
Jiang, 2017	Cohort	1	1	1	1	2	0	1	1	8
Jithin, 2016	cross‐sectional	1	1	1	1	0	1	0	NA	5
Jurjans, 2019	Cohort	0	1	1	1	0	0	1	1	5
Lim, 2015	Cohort	1	1	1	1	2	1	1	1	9
Magwood, 2017	Cross‐sectional	1	0	0	0	1	1	1	NA	4
Kothagundla, 2021	Cohort	1	1	1	1	2	0	1	0	7
Mechtouff, 2018	Cross‐sectional	1	0	0	2	2	1	1	NA	7
Park, 2017	Cohort	1	1	1	1	2	0	1	1	8
Rijkmans, 2018	Cohort	0	1	1	1	1	1	1	1	7
Sarfo, 2016	Cohort	1	1	1	1	2	0	1	0	7
Sluggett, 2015	Cohort	1	1	1	1	2	0	1	1	8
Ullberg, 2017	Cohort	1	1	1	1	1	0	1	0	6
Yeo, 2020	Cohort	1	1	1	1	2	1	1	1	9
Preinreich, 2021	Cohort	1	1	1	1	2	1	1	0	8
Rodríguez‐Bernal, 2021	Cohort	1	1	1	1	2	1	1	0	8
Sheehan, 2021	Cohort	1	1	1	1	2	1	1	0	8
Tiili, 2021	Cohort	1	1	1	1	2	1	1	1	9
Guidoux, 2019	Cohort	0	1	1	1	2	0	1	1	7
Haeusler, 2015	Cross‐sectional	1	0	1	0	2	1	1	NA	6
Mazurek, 2017	Cohort	1	1	1	1	2	0	1	1	8
Sauer, 2015	Cohort	0	1	1	1	2	0	1	1	7
Shah, 2016	Cohort	1	1	1	1	2	1	1	1	9
Xian, 2015	Cohort	1	1	1	1	2	0	1	1	8
Xu, 2017	Cohort	0	1	1	1	2	1	1	0	7
Abanto, 2020	Cross‐sectional	1	0	0	2	2	2	1	NA	8
Chang, 2020	Cohort	1	1	1	1	2	1	1	1	9
Dalli, 2020	Cohort	1	1	1	1	2	0	1	1	8
Zhang, 2017	Cross‐sectional	1	1	0	2	2	2	1	NA	9
Flach, 2020	Cohort	1	1	1	1	2	1	1	1	9
Gronemann, 2020	Cohort	1	1	1	1	2	1	1	1	9
Gynnild, 2020	Cohort	1	1	1	1	2	1	1	1	9
MacDonald, 2020	Cross‐sectional	1	0	0	1	1	2	1	NA	6
Malaeb, 2020	Cross‐sectional	1	0	0	1	0	2	1	NA	5
Saade, 2021	Cross‐sectional	1	0	0	2	0	1	1	NA	5
Shankari, 2020	Cross‐sectional	1	0	0	2	2	1	1	NA	7
Yeo, 2020	Cross‐sectional	1	0	1	2	2	2	1	NA	9

### 3.1 Antiplatelet medications

#### 3.1.1 Prescription rate of antiplatelet medications

**FIGURE 1:**
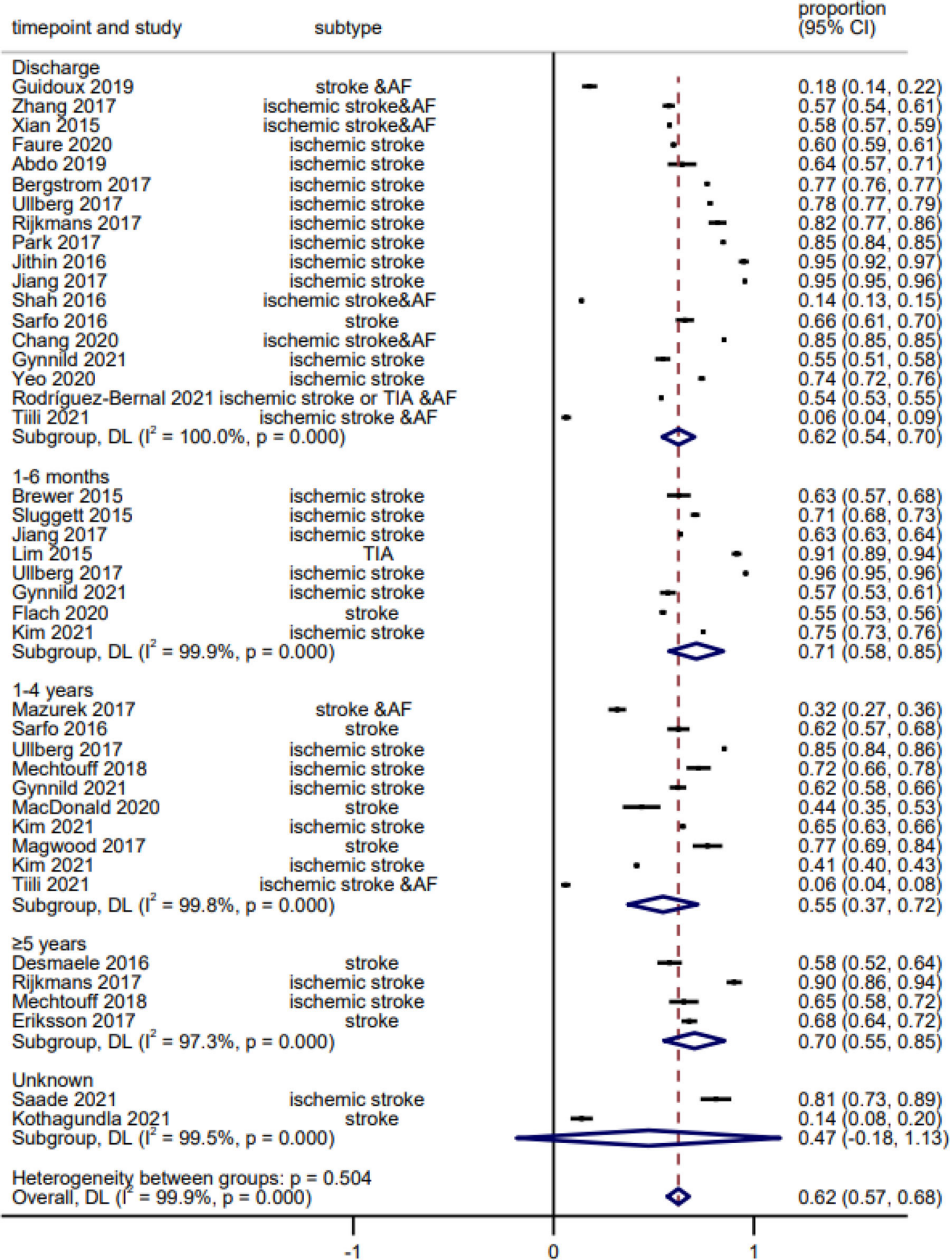
Forest plot of prescribed antiplatelet medications

The pooled proportion of prescribed antiplatelet medication is 62% (95% CI: 57%−68%) (Figure 1), with 62% (95% CI: 54%−70%), 71% (95% CI: 58%−85%), 55% (95% CI: 37%−72%), and 70% (95% CI: 55%−85%) at discharge, 1–6 months, 1–4 years, and 5 years post index stroke, respectively (Figure 1).

#### 3.1.2 Medication adherence

**FIGURE 2:**
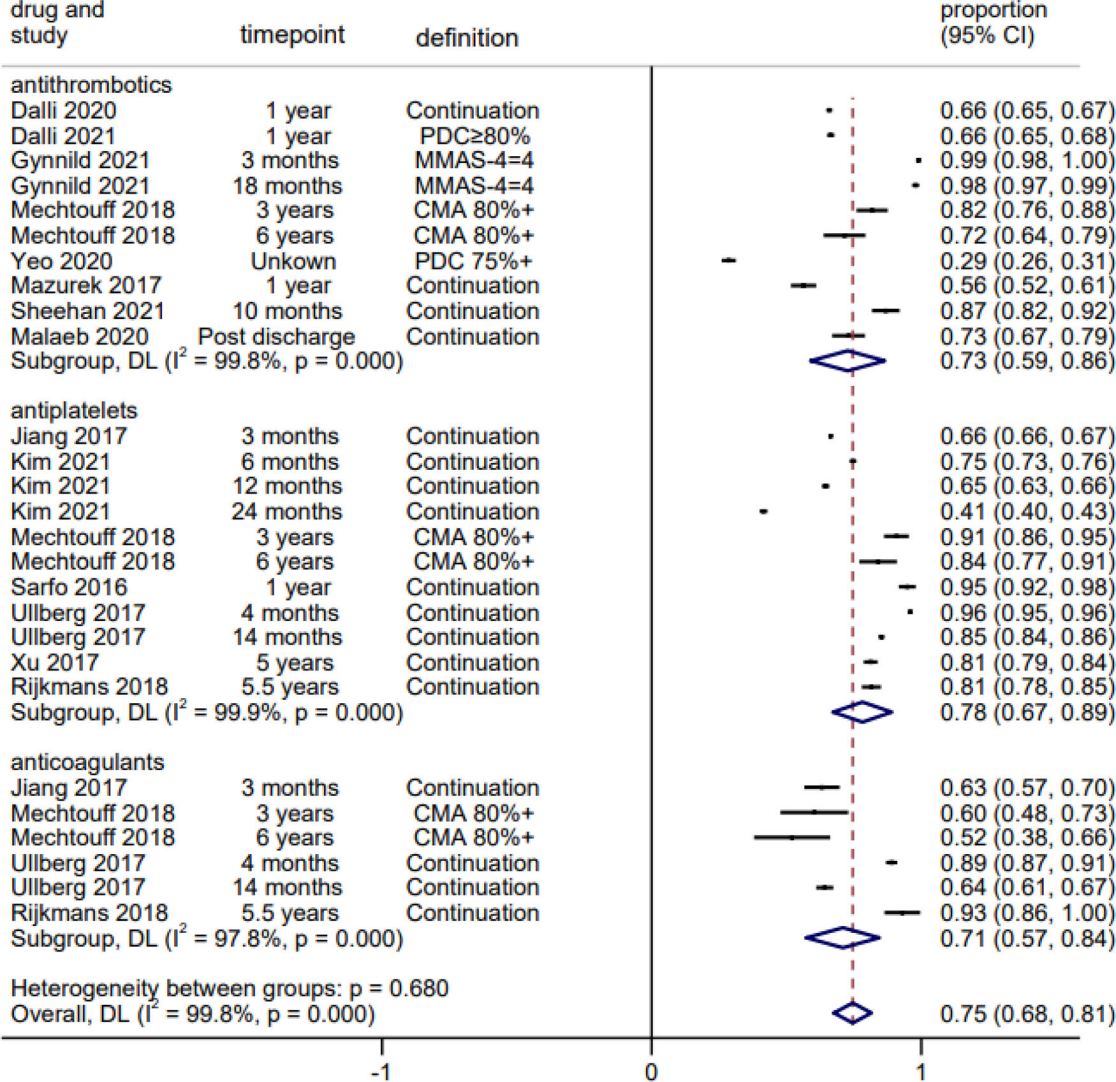
Forest plot of medication adherence

Definitions for adherence are heterogeneous between studies (Table 3). Good adherence to antiplatelet is 78% (95% CI: 67%−89%) (Figure 2). The adherence rate is 79% (95% CI: 64%−95%), 72% (95% CI: 39%−106%), and 82% (95% CI: 80%−84%) for ≤1, 1–4, and ≥5 years post index stroke, respectively (Figure S2). Adherence reported by high‐income countries (HICs) (77%, 95% CI: 63%−91%) was lower than that in the low‐and‐middle‐income countries (LMICs) (81%, 95% CI: 64%−98%) (Figure S2).

**TABLE 3:** Adherence to antithrombotics medications

Study	Population	Time post index stroke	Definition	Findings
Mechtouff, 2018	IS or TIA	3 years and 6 years post index stroke	Continuous Measure of Medication Acquisition (CMA) was defined as medication adherence. CMA≥80%	Adherence to any antithrombotic drugs was 82% and 72%, at 3 years and 6 years, respectively.
				Adherence to anticoagulant was 60% and 52%, at 3 years and 6 years, respectively.
				Adherence to the antiplatelet drug was 91% and 84%, at 3 years and 6 years, respectively.
Xu, 2017	IS	5 years	Discontinuation of antiplatelet therapy	165 Discontinued during follow up
Yeo, 2020	IS	Unkown	Adherence was defined using PDC: high (≥75%), intermediate (50%−74%), low (25%−49%), and very low (< 25%).	29%, 18%, 20%, and 34% had high, intermediate, low, and very low adherence to antithrombotic medications, respectively.
Ullberg, 2017	IS	4 months	Primary drug adherence was defined as filling the first drug prescription within 120 days after stroke.	Drug adherence rates 4 months post‐stroke were 96% for antiplatelet drugs, and 90% for warfarin.
Ullberg, 2017	IS	14 months	Drug persistence at 14 months was defined as filling a prescription between 10 and 14 months after stroke.	Drug adherence rates 14 months post‐stroke were 85% for antiplatelet drugs, and 69% for warfarin.
Sarfo, 2016	Stroke	1 year	Persistence was defined as the continuation of medications.	Persistent rate was 95% for antiplatelets, and 50% for anticoagulants.
Jiang, 2017	IS or TIA	3 months	Three‐month persistence was defined as continuation of all secondary preventive medications prescribed at discharge.	Persistence at 3 months after discharge was 66.35% for antiplatelets, and 63.16% for warfarin.
Mazurek, 2017	Stroke & AF	1 year	Persistence was defined as the continuation of medications.	56% were adherent to antithrombotic treatment
Gynnild, 2021	Ischemic stroke	3 months	MMAS‐4 = 4 (high adherence)	469/474 (99%)
Gynnild, 2021	Ischemic stroke	18 months	MMAS‐4 = 4 (high adherence)	464/474 (98%)
Dalli, 2020	stroke or TIA	1 year	Discontinuation was assessed among medication users and defined as having no medication supply for ≥90 days in the year postdischarge.	2426/7112 (34.1)
Dalli, 2021	stroke or TIA	1 year	Adherence to each medication group was estimated using the proportion of days covered (PDC) method from hospital discharge until the 1‐year landmark date.	3218/4845 (66.4)
Malaeb, 2020	IS	Post discharge	Post discharge prescription medications.	149/204 (73%)
Kim, 2021	IS	6 months	Discontinuation was defined as when the antiplatelet agents were discontinued without refills throughout the rest of the observation period.	Prevalence of premature discontinuation of antiplatelets within 6 months was 25.3%
Kim, 2021	IS	12 months	Discontinuation was defined as when the antiplatelet agents were discontinued without refills throughout the rest of the observation period.	Prevalence of premature discontinuation of antiplatelets within 12 months was 35.5%
Kim, 2021	IS	24 months	Discontinuation was defined as when the antiplatelet agents were discontinued without refills throughout the rest of the observation period.	Prevalence of premature discontinuation of antiplatelets within 24 months was 58.5%
Rijkmans, 2018	IS	5.5 years	Discontinuation of medication was considered nonpersistent.	Persistent rate was 90% for aspirin, 72% for dipyridamole, and 53% for anticoagulants.
Sheehan, 2021	IS	10 months	Medication persistence was defined as the continuation of medication classes prescribed at hospital discharge.	Persistent rate was 87% for antithrombotics

AF: atrial fibrillation; IS: ischemic stroke; TIA: transient ischemic stroke.

### 3.2 Anticoagulant medications

#### 3.2.1 Prescription rate of anticoagulant medications

**FIGURE 3:**
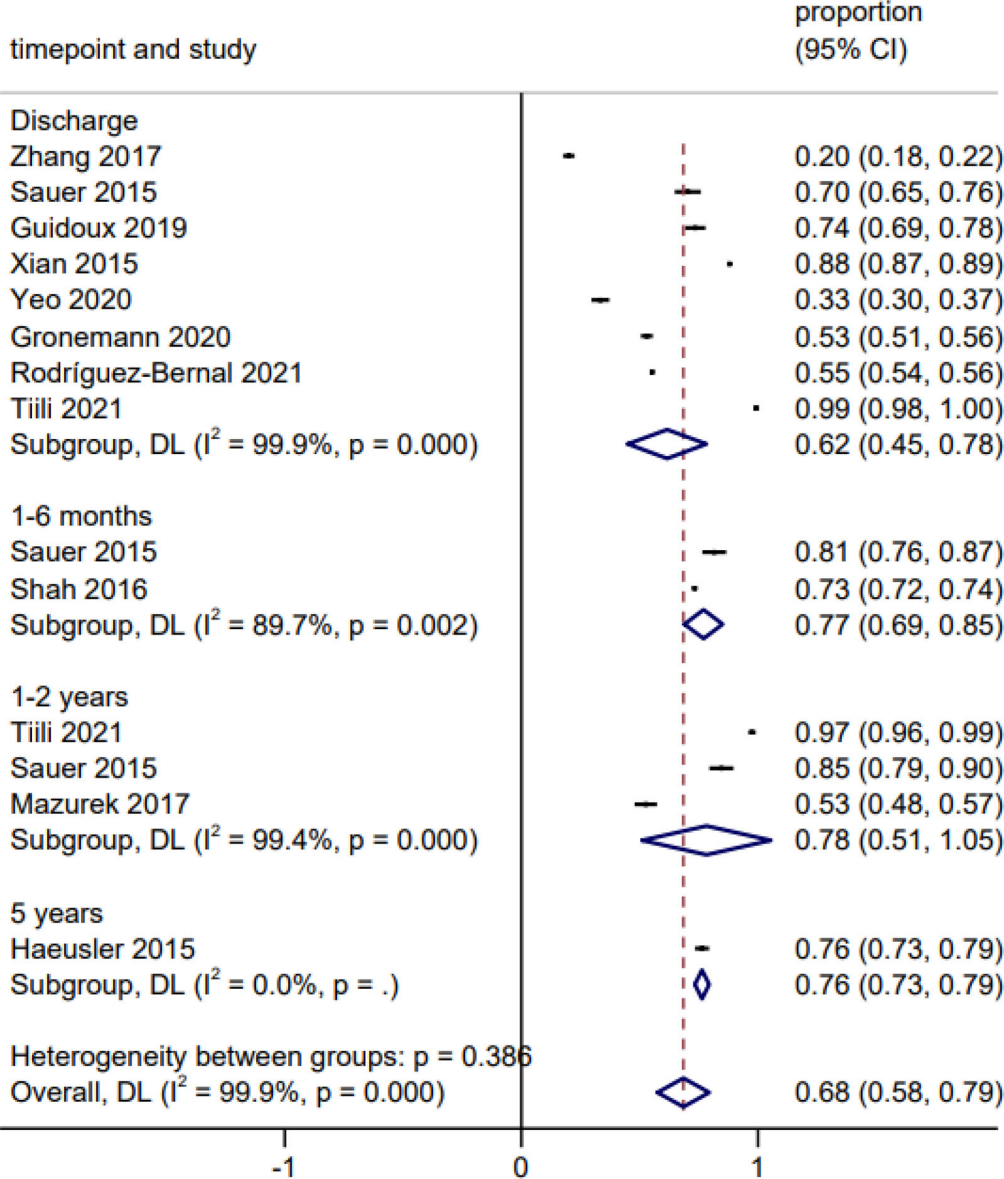
Forest plot of prescribed anticoagulants among patients with AF

The pooled proportion of prescribed anticoagulants among patients with AF is 68% (95% CI: 58%−79%) (Figure 3), with 62% (95% CI: 45%−78%), 77% (95% CI: 69%−85%), 78% (95% CI: 51%−105%), and 76% (95% CI: 73%−79%) at discharge, 1–6 months, 1–2 years, and 5 years post index stroke, respectively (Figure 3).

#### 3.2.2 Medication adherence

Good adherence to anticoagulant is 71% (95% CI: 57%−84%) (Figure 2). The adherence rate is 76% (95% CI: 51%−102%), 64% (95% CI: 61%−67%), and 73% (95% CI: 33%−113%) for ≤1, 1–4, and ≥5 years post index stroke, respectively (Figure S3). Adherence reported by HICs (73%, 95% CI: 57%−88%) was higher than that in the LMICs (63%, 95% CI: 57%−74%) (Figure S3).

### 3.3 Adherence to antithrombotic medications

Good adherence to antithrombotic medications is 73% (95% CI: 59%−86%) (Figure 2). Studies that defined adherence using prescription refill had higher adherence rate of 74% (95% CI: 55%−93%) than studies that used medication continuation: 70% (95% CI: 60%−81%) (Figure S4). The adherence rate is 75% (95% CI: 57%−93%), 90% (95% CI: 74%−106%), and 72% (95% CI: 64%−79%) for ≤1, 1–4, and ≥5 years post index stroke, respectively (Figure S4). Adherence reported by HICs (73%, 95% CI: 58%−87%) was approximate to that in the LMICs (73%, 95% CI: 67%−79%) (Figure S4).

### 3.4 Optimal treatment

**FIGURE 4:**
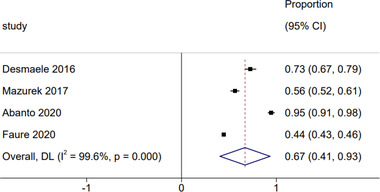
Forest plot of guideline antithrombotics

**FIGURE 5:**
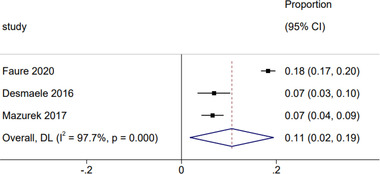
Forest plot of not receive any antithrombotic medications as recommended

The recommendations from major guidelines are summarized in table S1 (Coutts et al., 2014; Kleindorfer et al., 2021; Klijn et al., 2019; Liu et al., 2020; Ringleb et al., 2008;). The proportion of patients who were treated according to the recommendation of guidelines of antithrombotic medications is 67% (95% CI: 41%−93%) (Figure 4). 11% (95% CI: 2%−19%) of patients did not receive any antithrombotic medications as recommended (Figure 5). Faure et al. (2020) reported that 36% of patients received ≥2 antiplatelets or a combination of antiplatelet and anticoagulant. Such combinations are not recommended because of the potential increased risk of bleeding (Table S1).

## 4 DISCUSSION

In this systematic review and meta‐analysis, we summarized the proportions of antithrombotic medication prescription and adherence in patients with stroke. We found that less than 70% of patients were prescribed and treated according to the recommended guidelines of antithrombotic medications. Good adherence to antiplatelet, anticoagulant, and antithrombotic medications was 78% (95% CI: 67%−89%), 71% (95% CI: 57%−84%), and 73% (95% CI: 59%−86%), respectively. It was reported that 11% (95% CI: 2%−19%) of patients did not receive antithrombotic medications.

We found the lowest rates of anticoagulant prescription in Asia, compared with Europe and Americas (Figure S5), which is in line with a previous study (Kozieł et al., 2021). Moreover, our results show that prescription for antiplatelet medication is highest in Asia (Figure S6). This may be because large artery atherosclerosis was the leading ischemic stroke etiology in Asians and less anticoagulants were prescribed for Asian stroke patients with AF (Ornello et al., 2018).

In our results, the prescribing rate (68%) of anticoagulants for patients with AF has increased, compared to 45% in the past decade (Hsu et al., 2016). There are around only 50% of patients still taking anticoagulants therapy by 2 years in the past 5–10 years studies (Collings et al., 2017; Deitelzweig et al., 2013; Wang et al., 2016), whereas our statistical analysis showed that the good adherence rate is 64% for 1–4 years post index stroke. This may be due to promotion of the AF management guidelines, along with the improvement of educational and economic standards.

Although our prescription rate has increased from the previous decade, it is still less than 70%. We suspect that the insufficient prescription rate may still exist for the following reasons: uncertainty about clinical benefits and risks, knowledge and experience deficit, competing medical issues, and medication cost (Gross et al., 2003; Kirley et al., 2016). There may be potential ways to increase antithrombotic drug prescription rates, for example, increasing physicians' awareness of under‐treatment, emphasizing accurate assessment of bleeding risk (Hsu et al., 2016), and addressing drug high cost in some areas.

We also found lower adherence rate with anticoagulant in low‐ and middle‐income countries compared with that in high‐income countries, which may be related to different educational levels and cultural concepts. We found lower adherence rate with antiplatelet in high‐income countries compared with that in low‐ and middle‐income countries. This may be due to the fact that Asians are more afraid of the risk of bleeding from anticoagulants, so they prefer antiplatelet drugs, while patients in developed countries are the opposite (Lowres et al., 2019). Given the association of nonadherence with increased morbidity and mortality (Viswanathan et al., 2012), adequate measures taken to improve medication adherence should receive much more attention in stroke patients. These strategies can be: (1) medical insurance or medication cost was associated with medication adherence (Kronish et al., 2013; Wang et al., 2006) as reducing drug costs or increasing health insurance coverage may increase medication adherence; 2) large‐scale, national public health campaigns to focus on groups of medications effective for secondary prevention in stroke may make patients or caregivers take notice; and 3) patient education regarding medications to improve adherence. Then regular follow‐up visits and direct asking about medication adherence could be efficient.

There are several limitations in this meta‐analysis. First, there is no common gold standard method for evaluating medication adherence, which may introduce measurement bias in our results. Second, the pooling data were highly heterogeneous; this was not explained by differences in patient characteristics. We conducted subgroup analyses to pool the same definitions, study design, country, and timepoint; however, residual heterogeneity persisted.

## 5 CONCLUSION

In conclusion, we found that less than 70% of patients were prescribed and treated according to the recommended guidelines of antithrombotic medications, and good adherence to antithrombotic medications is only 73%. Prescription rate and good adherence to antithrombotic medications still need to be improved among stroke survivors.

## CONFLICT OF INTEREST

The authors declare no conflict of interest.

### PEER REVIEW

The peer review history for this article is available at https://publons.com/publon/10.1002/brb3.2752

## Supporting information

Appendix Search strategy

Figure S1. Flow chart of the systematic review and meta‐analysis
Figure S2. Forest plot of Subgroup analyses of antiplatelet medication adherence
Figure S3. Forest plot of Subgroup analyses of anticoagulants adherence among patients with AF
Figure S4. Forest plot of Subgroup analyses of antithrombotic adherence
Figure S5. Forest plot of Subgroup analyses of prescribed anticoagulants among patients with AFSfigure 6. Forest plot of Subgroup analyses of prescribed antiplatelet medications

Table S1. Recommendations for secondary stroke prevention according to major guidelines

## Data Availability

All data included in this study are available upon request by contact with the corresponding author.
