# Supplementary material for: Antithrombotics prescription and adherence among stroke survivors: A systematic review and meta‐analysis
Source: Brain Behav. 2022 Sep 6;12(10):e2752. doi: 10.1002/brb3.2752 (PMC9575604; doi:10.1002/brb3.2752)
Supplement: Supplementary file 1 — Appendix Search strategy [file BRB3-12-e2752-s002.docx]

Search strategy

pubmed

**/*stroke*/**

1. cerebrovascular disorders/ or basal ganglia cerebrovascular disease/ or brain ischemia/ or carotid artery diseases/ or intracranial arterial diseases/ or "intracranial embolism and thrombosis"/ or intracranial hemorrhages/ or stroke/ or brain infarction/ or vasospasm, intracranial/ or vertebral artery dissection/

2. (stroke or cerebrovasc* or brain vasc* or cerebral vasc* or cva* or apoplex* or isch?emi* attack* or tia).tw.

3. ((brain* or cerebr* or cerebell* or hemispher* or intracran* or intracerebral or infratentorial or supratentorial or MCA) and (isch?emi* or infarct* or thrombo* or emboli* or occlus* or hypox* or vasospasm)).tw.

4. ((brain* or cerebr* or cerebell* or intracerebral or intracran* or intraventricular or infratentorial or supratentorial or basal gangli*) and (haemorrhage* or hemorrhage* or haematoma* or hematoma* or bleed*)).tw.

5. 1 or 2 or 3 or 4

**/*Antiplatelet therapy*/**

6. Platelet aggregation inhibitors/

7. Cyclooxygenase Inhibitors/ or Thienopyridines/ or Phosphodiesterase Inhibitors/ or Thromboxane A2/ or Purinergic P2Y Receptor Antagonists/

8. Platelet activation/

9. Blood platelets/

10. (antiplatelet* antithrombocytic).tw.

11. ((platelet* or thrombocyte*) and (inhibit* or antagonist* or antiaggreg*)).tw.

12. (cyclooxygenase inhibitor* or thienopyridine* or phosphodiesterase inhibitor*).tw.

13. (thromboxane A2 and (inhib* or antag*)).tw.

14. (aspirin* or acetyl salicylic acid* or acetyl?salicylic acid*).tw.

15. (ARC1779 or AZD6140 or alprostadil or asasantin or carnitine or cilostazol or clopidogrel or cloricromene or cv4151 or defibrotide or dilazep or dipyridamol* or disintegrin* or ditazol or E5880 or E5510 or epoprostenol* or fluribrofen or fut175 or iloprost* or indobufen or isbogrel or kbt3022 or ketanserin* or ketoprofen or ketorolac or levamisol* or ligustrazine* or tromethamine* or milrinone* or mopidamol* or naudicelle or nimesulide or ozagrel* or oky046 or phthalzinol or picotamide or policosanol or prasugrel or procainamide or sarpogrelate or satigrel or sulphinpyrazone or sulfinpyrazone or suloctadil or terutroban or ticagrelor or ticlopidine or trapidil or triflusal or vorapaxar).tw.

16. or/6‐15

**/*Anticoagulant therapy*/**

17. anticoagulants/
18. Blood coagulation factors/ or Blood coagulation/
19. (anticoagul* or antithromb*).tw.
20. Warfarin/ or 4 hydroxycoumarins/ or acenocoumarol/ or coumarins/ or dicumarol/ or ethyl biscoumacetate/ or phenindione/ or phenprocoumon/
21. Vitamin K/
22. (warfarin* or coumadin* or coumarin* or cumarin* or phenprocoum* or phenprocum* or dicoumar* or dicumar* or acenocoumar* or acenocumar* or fluindione or phenindione or clorindione or diphenadione or ethyl biscoumacetate).tw.
23. (Vitamin K antagonist* or VKA or VKAs or antivitamin K).tw.
24. antithrombins/ or hirudin therapy/ or thrombin/
25. ((direct* and thrombin and inhib*) or DTI?).tw.
26. (argatroban or MD805 or dabigatran or ximelagatran or melagatran or efegatran or flovagatran or inogatran or napsagatran or bivalirudin or lepirudin or hirudin* or desirudin or desulfatohirudin or hirugen or hirulog or AZD0837 or bothrojaracin or odiparcil).tw.
27. factor Xa/
28. ((factor Xa or factor 10a or fXa or autoprothrombin c or thrombokinase) and inhib*).tw.
29. (activated and (factor X or factor 10) and inhib*).tw.
30. xabans.tw.
31. (antistasin or apixaban or betrixaban or du 176b or eribaxaban or fondaparinux or idraparinux or otamixaban or razaxaban or rivaroxaban or yagin or ym 150 or ym150 or LY517717 or darexaban or edoxaban or SSR126517E or fidexaban or idrabiotaparinux or letaxaban or tanogitran or taxexaban).tw.
32. heparin/ or heparin, low molecular weight/ or heparinoids/
33. (heparin* or lmwh* or enoxaparin* or glycosaminoglycan* or nadroparin* or mesoglycan* or tedelparin* or certoparin or tinzaparin or parnaparin or dalteparin or reviparin or fraxiparin* or danaparoid or lomoparan or org 10172 or mesoglycan or pentosan polysul* or sp54 or cy222 or cy216 or dermatan sul* or heparan sul*).tw.
34. or/17‐33

35. 16 or 34

/***Study type***/

36. Epidemiologic studies/ or cohort studies/ or (cohort and (study or studies)).tw. or Cohort analy*.tw. or (Follow up and (study or studies)).tw. or (observational and (study or studies)).tw. or Longitudinal.tw. or Retrospective.tw. or Cross sectional.tw. or Cross sectional studies

/*limit to humans*/

37. 5 and 35 and 36
38. (animals/ or invertebrate/ or animal experiment/ or animal model/ or animal tissue/ or animal cell/ or nonhuman/) not (human/ or normal human/ or human cell/)
39. 37 not 38

embase

**/*stroke*/**

1. 'cerebrovascular disorders' OR 'basal ganglia cerebrovascular disease'/exp OR 'brain ischemia'/exp OR 'carotid artery diseases'/exp OR 'intracranial arterial diseases'/exp OR 'intracranial embolism and thrombosis'/exp OR 'intracranial hemorrhages'/exp OR stroke OR 'brain infarction'/exp OR 'vasospasm, intracranial' OR 'vertebral artery dissection'

2. stroke:ti,ab,kw OR cerebrovasc*:ti,ab,kw OR 'brain vasc*':ti,ab,kw OR 'cerebral vasc*':ti,ab,kw OR cva*:ti,ab,kw OR apoplex*:ti,ab,kw OR 'isch?emi* attack*':ti,ab,kw OR tia*1:ti,ab,kw

3. (brain*:ti,ab,kw OR cerebr*:ti,ab,kw OR cerebell*:ti,ab,kw OR hemispher*:ti,ab,kw OR intracran*:ti,ab,kw OR intracerebral:ti,ab,kw OR infratentorial:ti,ab,kw OR supratentorial:ti,ab,kw OR mca:ti,ab,kw) AND (isch?emi*:ti,ab,kw OR infarct*:ti,ab,kw OR thrombo*:ti,ab,kw OR emboli*:ti,ab,kw OR occlus*:ti,ab,kw OR hypox*:ti,ab,kw OR vasospasm:ti,ab,kw)

4. (brain*:ti,ab,kw OR cerebr*:ti,ab,kw OR cerebell*:ti,ab,kw OR intracerebral:ti,ab,kw OR intracran*:ti,ab,kw OR intraventricular:ti,ab,kw OR infratentorial:ti,ab,kw OR supratentorial:ti,ab,kw OR 'basal gangli*':ti,ab,kw) AND (haemorrhage*:ti,ab,kw OR hemorrhage*:ti,ab,kw OR haematoma*:ti,ab,kw OR hematoma*:ti,ab,kw OR bleed*:ti,ab,kw)

5. 1 or 2 or 3 or 4

**/*Antiplatelet therapy*/**

6. 'platelet aggregation inhibitors'/exp

7. 'cyclooxygenase inhibitors'/exp OR 'thienopyridines'/exp OR 'phosphodiesterase inhibitors'/exp OR 'thromboxane a2/ai' OR 'purinergic p2y receptor antagonists'/exp

8. 'platelet activation'/exp

9. 'blood platelets'/exp

10. 'antiplatelet* antithrombocytic':ti,ab,kw

11. (platelet*:ti,ab,kw OR thrombocyte*:ti,ab,kw) AND (inhibit*:ti,ab,kw OR antagonist*:ti,ab,kw OR antiaggreg*:ti,ab,kw)

12. 'cyclooxygenase inhibitor*':ti,ab,kw OR thienopyridine*:ti,ab,kw OR 'phosphodiesterase inhibitor*':ti,ab,kw

13. 'thromboxane a2':ti,ab,kw AND (inhib*:ti,ab,kw OR antag*:ti,ab,kw)

14. aspirin*:ti,ab,kw OR 'acetyl salicylic acid*':ti,ab,kw OR 'acetyl?salicylic acid*':ti,ab,kw

15. arc1779:ti,ab,kw OR azd6140:ti,ab,kw OR alprostadil:ti,ab,kw OR asasantin:ti,ab,kw OR carnitine:ti,ab,kw OR cilostazol:ti,ab,kw OR clopidogrel:ti,ab,kw OR cloricromene:ti,ab,kw OR cv4151:ti,ab,kw OR defibrotide:ti,ab,kw OR dilazep:ti,ab,kw OR dipyridamol*:ti,ab,kw OR disintegrin*:ti,ab,kw OR ditazol:ti,ab,kw OR e5880:ti,ab,kw OR e5510:ti,ab,kw OR epoprostenol*:ti,ab,kw OR fluribrofen:ti,ab,kw OR fut175:ti,ab,kw OR iloprost*:ti,ab,kw OR indobufen:ti,ab,kw OR isbogrel:ti,ab,kw OR kbt3022:ti,ab,kw OR ketanserin*:ti,ab,kw OR ketoprofen:ti,ab,kw OR ketorolac:ti,ab,kw OR levamisol*:ti,ab,kw OR ligustrazine*:ti,ab,kw OR tromethamine*:ti,ab,kw OR milrinone*:ti,ab,kw OR mopidamol*:ti,ab,kw OR naudicelle:ti,ab,kw OR nimesulide:ti,ab,kw OR ozagrel*:ti,ab,kw OR oky046:ti,ab,kw OR phthalzinol:ti,ab,kw OR picotamide:ti,ab,kw OR policosanol:ti,ab,kw OR prasugrel:ti,ab,kw OR procainamide:ti,ab,kw OR sarpogrelate:ti,ab,kw OR satigrel:ti,ab,kw OR sulphinpyrazone:ti,ab,kw OR sulfinpyrazone:ti,ab,kw OR suloctadil:ti,ab,kw OR terutroban:ti,ab,kw OR ticagrelor:ti,ab,kw OR ticlopidine:ti,ab,kw OR trapidil:ti,ab,kw OR triflusal:ti,ab,kw OR vorapaxar:ti,ab,kw

16. or/6‐15

**/*Anticoagulant therapy*/**

17. 'anticoagulants'/exp
18. 'blood coagulation factors'/exp OR 'blood coagulation'/exp
19. anticoagul*:ti,ab,kw OR antithromb*:ti,ab,kw
20. warfarin OR '4 hydroxycoumarins' OR acenocoumarol OR coumarins OR dicumarol OR 'ethyl biscoumacetate' OR phenindione OR phenprocoumon
21. 'vitamin k'/exp
22. warfarin*:ti,ab,kw OR coumadin*:ti,ab,kw OR coumarin*:ti,ab,kw OR cumarin*:ti,ab,kw OR phenprocoum*:ti,ab,kw OR phenprocum*:ti,ab,kw OR dicoumar*:ti,ab,kw OR dicumar*:ti,ab,kw OR acenocoumar*:ti,ab,kw OR acenocumar*:ti,ab,kw OR fluindione:ti,ab,kw OR phenindione:ti,ab,kw OR clorindione:ti,ab,kw OR diphenadione:ti,ab,kw OR 'ethyl biscoumacetate':ti,ab,kw
23. 'vitamin k antagonist*':ti,ab,kw OR vka:ti,ab,kw OR vkas:ti,ab,kw OR 'antivitamin k':ti,ab,kw
24. 'antithrombins'/exp OR 'hirudin therapy'/exp OR thrombin
25. direct*:ti,ab,kw AND thrombin:ti,ab,kw AND inhib*:ti,ab,kw OR dti*1:ti,ab,kw
26. argatroban:ti,ab,kw OR md805:ti,ab,kw OR dabigatran:ti,ab,kw OR ximelagatran:ti,ab,kw OR melagatran:ti,ab,kw OR efegatran:ti,ab,kw OR flovagatran:ti,ab,kw OR inogatran:ti,ab,kw OR napsagatran:ti,ab,kw OR bivalirudin:ti,ab,kw OR lepirudin:ti,ab,kw OR hirudin*:ti,ab,kw OR desirudin:ti,ab,kw OR desulfatohirudin:ti,ab,kw OR hirugen:ti,ab,kw OR hirulog:ti,ab,kw OR azd0837:ti,ab,kw OR bothrojaracin:ti,ab,kw OR odiparcil:ti,ab,kw
27. 'factor xa'
28. ('factor xa':ti,ab,kw OR 'factor 10a':ti,ab,kw OR fxa:ti,ab,kw OR 'autoprothrombin c':ti,ab,kw OR thrombokinase:ti,ab,kw) AND inhib*:ti,ab,kw
29. activated:ti,ab,kw AND ('factor x':ti,ab,kw OR 'factor 10':ti,ab,kw) AND inhib*:ti,ab,kw
30. xabans:ti,ab,kw
31. antistasin:ti,ab,kw OR apixaban:ti,ab,kw OR betrixaban:ti,ab,kw OR 'du 176b':ti,ab,kw OR eribaxaban:ti,ab,kw OR fondaparinux:ti,ab,kw OR idraparinux:ti,ab,kw OR otamixaban:ti,ab,kw OR razaxaban:ti,ab,kw OR rivaroxaban:ti,ab,kw OR yagin:ti,ab,kw OR 'ym 150':ti,ab,kw OR ym150:ti,ab,kw OR ly517717:ti,ab,kw OR darexaban:ti,ab,kw OR edoxaban:ti,ab,kw OR ssr126517e:ti,ab,kw OR fidexaban:ti,ab,kw OR idrabiotaparinux:ti,ab,kw OR letaxaban:ti,ab,kw OR tanogitran:ti,ab,kw OR taxexaban:ti,ab,kw
32. heparin OR 'heparin, low molecular weight'/exp OR heparinoids
33. heparin*:ti,ab,kw OR lmwh*:ti,ab,kw OR enoxaparin*:ti,ab,kw OR glycosaminoglycan*:ti,ab,kw OR nadroparin*:ti,ab,kw OR mesoglycan*:ti,ab,kw OR tedelparin*:ti,ab,kw OR certoparin:ti,ab,kw OR tinzaparin:ti,ab,kw OR parnaparin:ti,ab,kw OR dalteparin:ti,ab,kw OR reviparin:ti,ab,kw OR fraxiparin*:ti,ab,kw OR danaparoid:ti,ab,kw OR lomoparan:ti,ab,kw OR 'org 10172':ti,ab,kw OR mesoglycan:ti,ab,kw OR 'pentosan polysul*':ti,ab,kw OR sp54:ti,ab,kw OR cy222:ti,ab,kw OR cy216:ti,ab,kw OR 'dermatan sul*':ti,ab,kw OR 'heparan sul*':ti,ab,kw
34. or/17‐33

35. 16 or 34

/***Study type***/

36. 'Epidemiologic studies' or 'cohort studies'/exp or (cohort:ti,ab,kw AND (study:ti,ab,kw OR studies:ti,ab,kw)) or 'cohort analy*':ti,ab,kw or ('follow up':ti,ab,kw AND (study:ti,ab,kw OR studies:ti,ab,kw)) or (observational:ti,ab,kw AND (study:ti,ab,kw OR studies:ti,ab,kw)) or longitudinal:ti,ab,kw or Retrospective:ti,ab,kw or 'Cross sectional':ti,ab,kw or 'Cross sectional studies'

/*limit to humans*/

37. 5 and 35 and 36
38. (animals/exp or invertebrate/exp or 'animal experiment' or 'animal model' or 'animal tissue' or 'animal cell' or nonhuman) not (human or 'normal human' or 'human cell')

39. 37 not 38
