# Supplementary material for: Antithrombotics prescription and adherence among stroke survivors: A systematic review and meta‐analysis
Source: Brain Behav. 2022 Sep 6;12(10):e2752. doi: 10.1002/brb3.2752 (PMC9575604; doi:10.1002/brb3.2752)
Supplement: Supplementary file 2 — Figure S1. Flow chart of the systematic review and meta‐analysis Figure S2. Forest plot of Subgroup analyses of antiplatelet medication adherence Figure S3. Forest plot of Subgroup analyses of anticoagulants adherence among patients with AF Figure S4. Forest plot of Subgroup analyses of antithrombotic adherence Figure S5. Forest plot of Subgroup analyses of prescribed anticoagulants among patients with AFSfigure 6. Forest plot of Subgroup analyses of prescribed antiplatelet medications [file BRB3-12-e2752-s001.pdf]

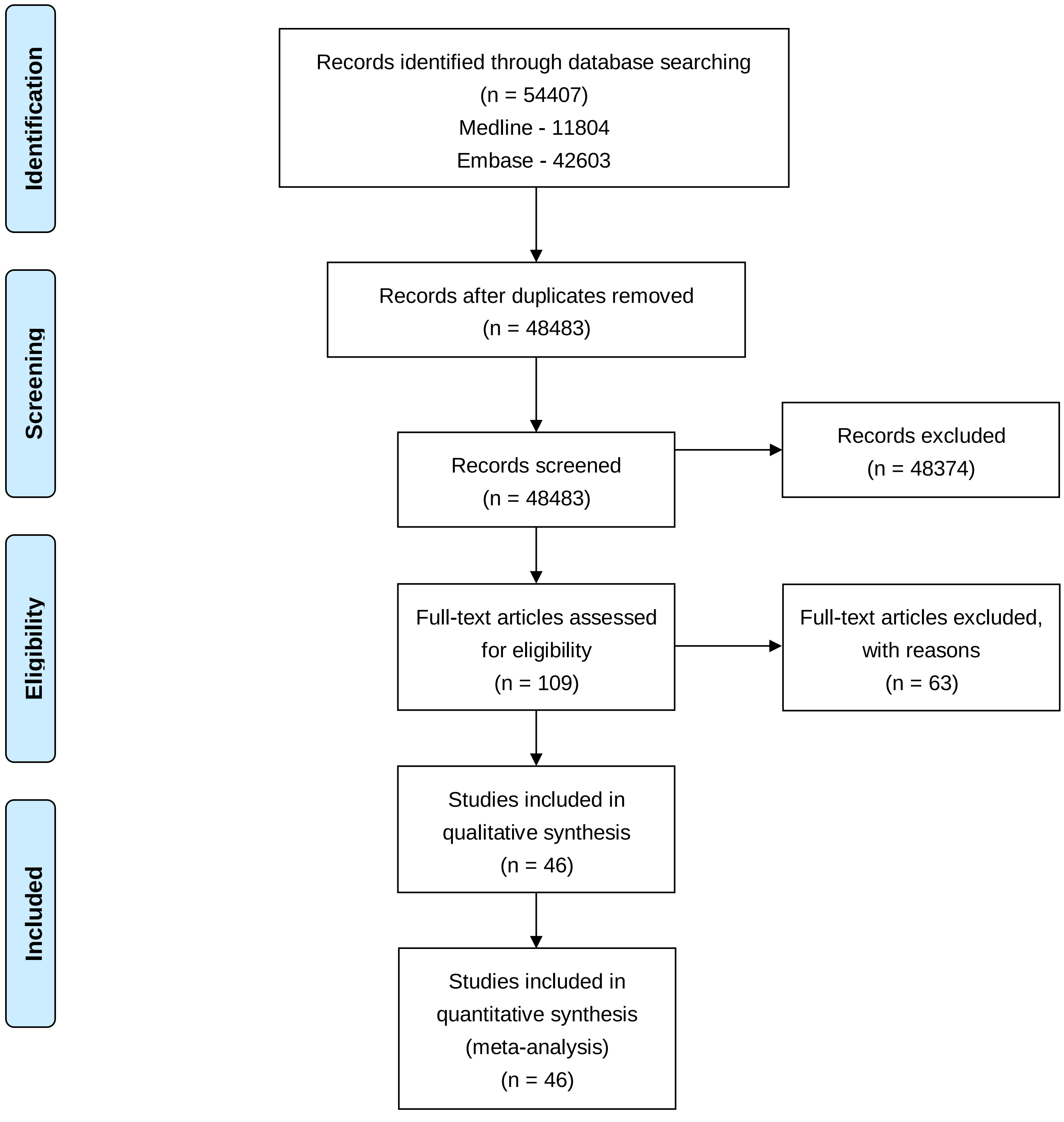


Sfigure 1 Flow chart of the systematic review and meta-analysis

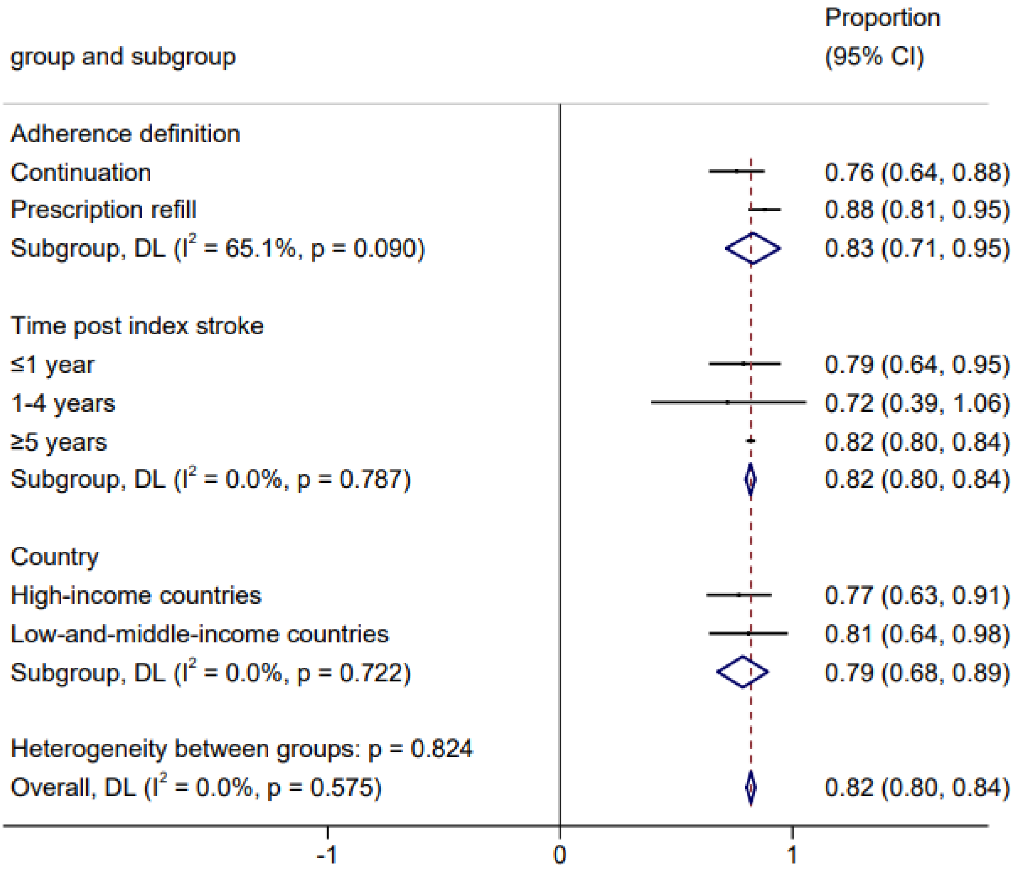


Sfigure 2 Forest plot of Subgroup analyses of antiplatelet medication adherence

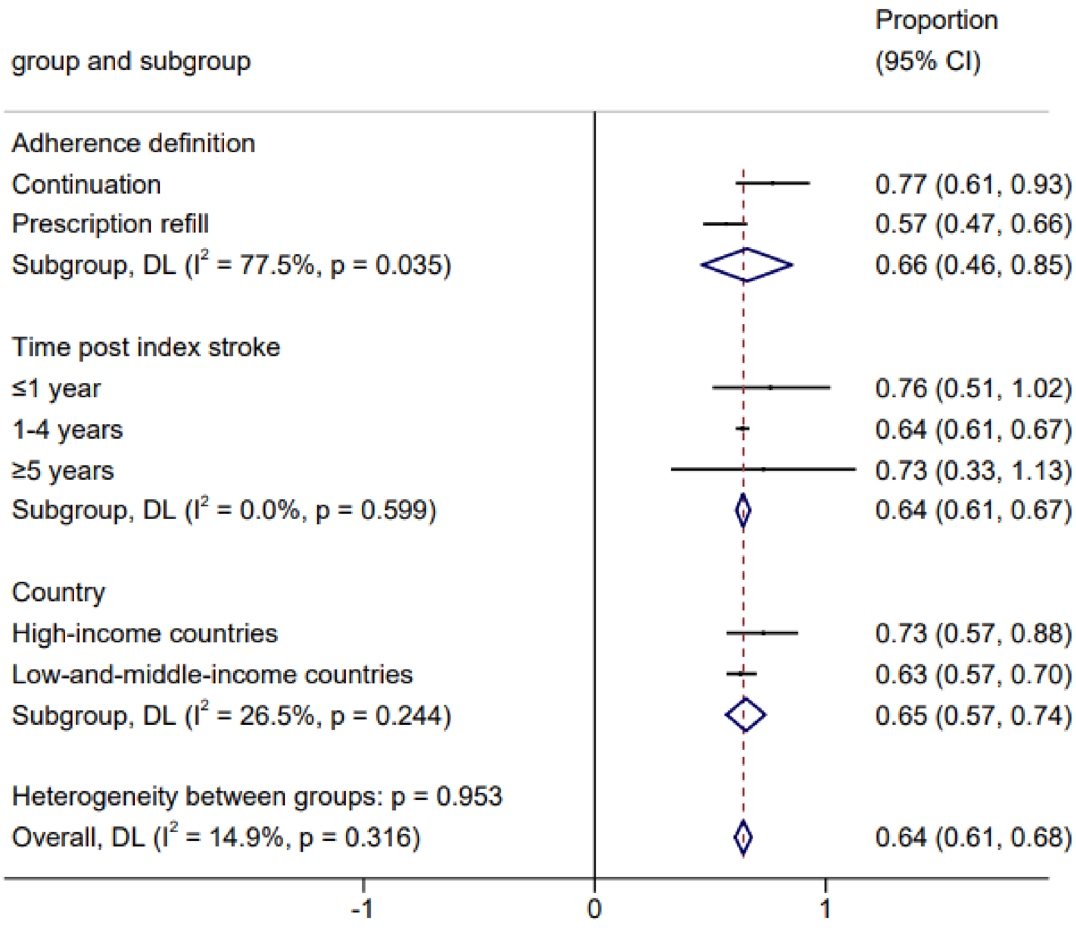


Sfigure 3 Forest plot of Subgroup analyses of anticoagulants adherence among patients with AF

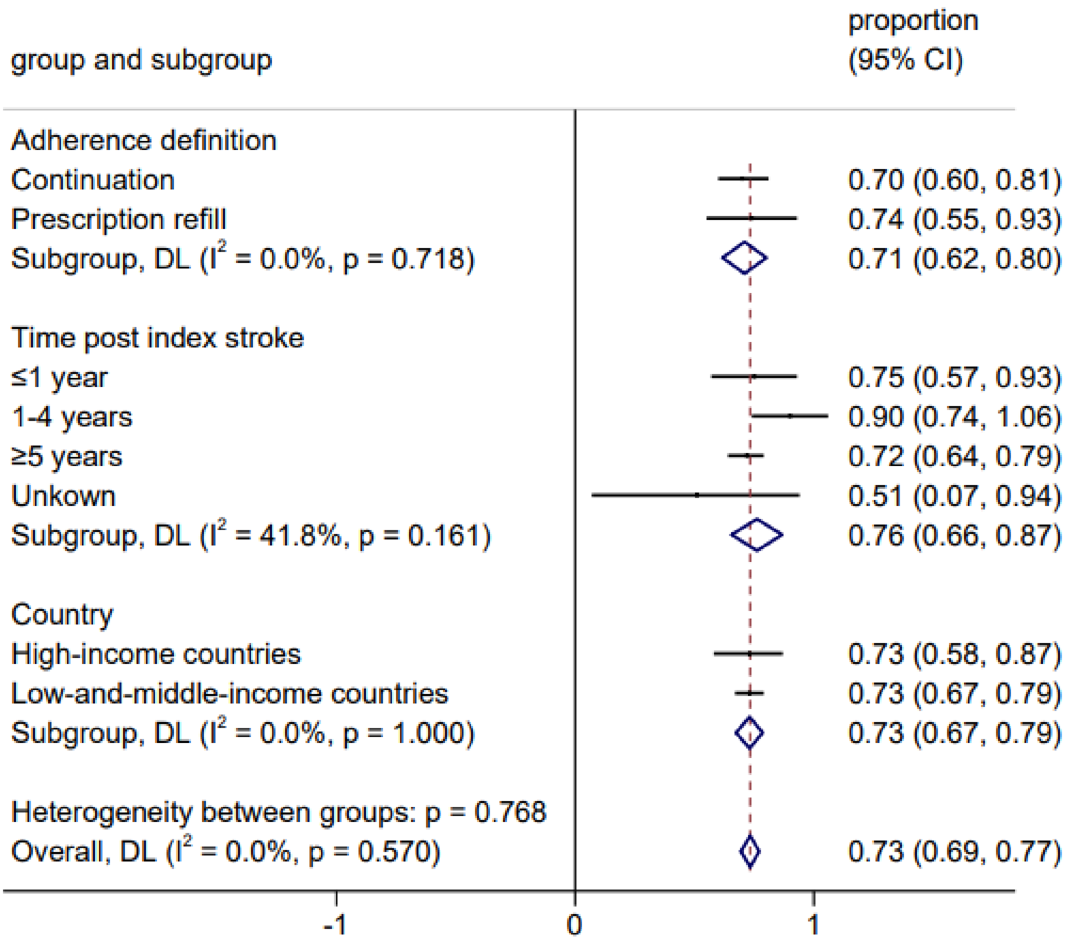


Sfigure 4 Forest plot of Subgroup analyses of antithrombotic adherence

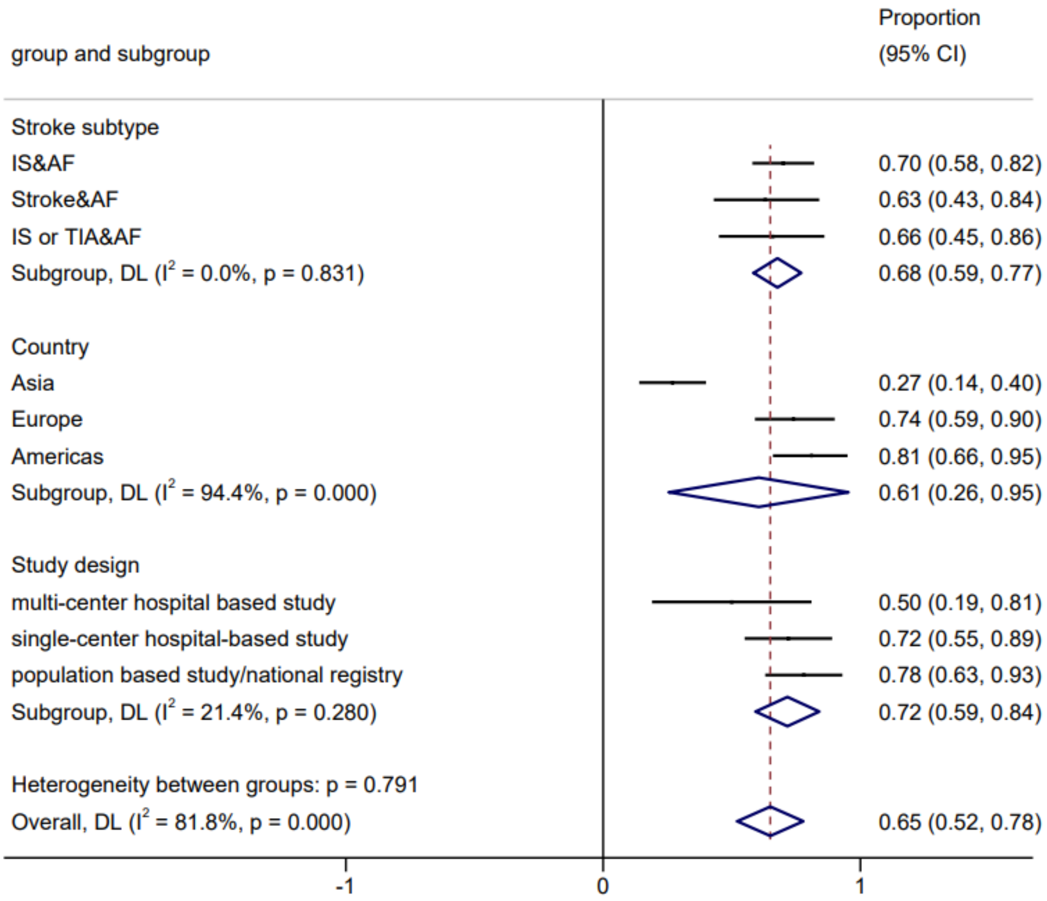


Sfigure 5 Forest plot of Subgroup analyses of prescribed anticoagulants among patients with AF

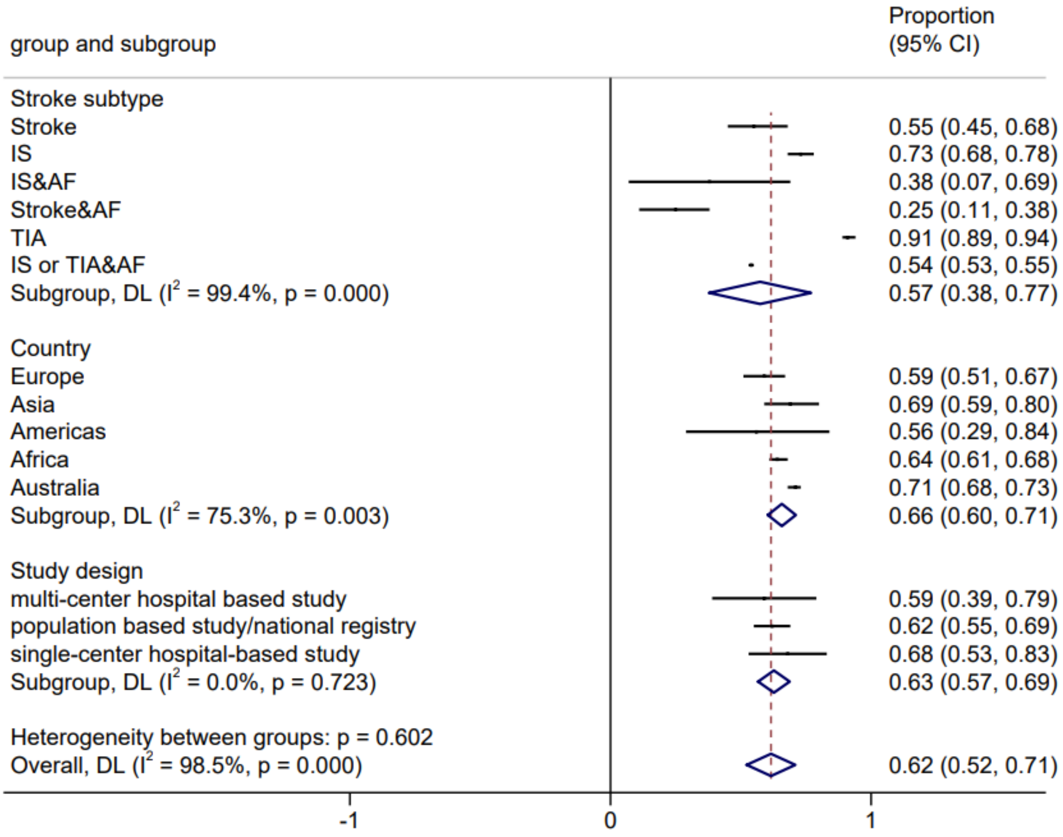


Sfigure 6 Forest plot of Subgroup analyses of prescribed antiplatelet medications
