# Supplementary material for: Antithrombotics prescription and adherence among stroke survivors: A systematic review and meta‐analysis
Source: Brain Behav. 2022 Sep 6;12(10):e2752. doi: 10.1002/brb3.2752 (PMC9575604; doi:10.1002/brb3.2752)
Supplement: Supplementary file 3 — Table S1. Recommendations for secondary stroke prevention according to major guidelines [file BRB3-12-e2752-s003.docx]

**Stable 1 Recommendations for secondary stroke prevention according to major guidelines**

|  | Canada 2014 (77) | AHA/ASA 2021 (78) | ESO 2008 (79) | China 2020 (80) |
| --- | --- | --- | --- | --- |
| Antiplatelet therapy | all patients with AIS should receive an antiplatelet therapy. | Antithrombotic therapy, including antiplatelet or anticoagulant agents, is recommended for nearly all stroke patients without contraindications. | Patients not requiring anticoagulation should receive antiplatelet therapy.  In patients with non-valvular AF and previous IS or TIA, we do not recommend antiplatelet agents (81). | For patients with symptomatic intracranial artery stenosis, antiplatelet therapy should be started as soon as possible and used for long term. |
| Anticoagulant therapy | anticoagulation is, however, recommended in the presence of concomitant AF. | Anticoagulation is usually recommended if the patient with IS or TIA and AF has no contraindications. | In patients with AF and previous stroke or TIA, oral anticoagulants reduce the risk of recurrence over antiplatelets or no antithrombotic treatment.  Non-vitamin K antagonist oral anticoagulants are preferred over vitamin K antagonists (81). | For patients with ischemic stroke or TIA with atrial fibrillation, appropriate doses of warfarin are recommended to prevent the recurrence of thromboembolism. |

AIS: acute ischemic stroke; TIA: transient ischemic stroke, IS: ischaemic stroke, AF: atrial fibrillation, AHA/ASA: American Heart Association/American Stroke Association, ESO: European Stroke Organization

References

77. Coutts SB, Wein TH, Lindsay MP, Buck B, Cote R, Ellis P, et al. Canadian Stroke Best Practice Recommendations: secondary prevention of stroke guidelines, update 2014. Int J Stroke. 2015;10(3):282-91.

78. Kleindorfer DO, Towfighi A, Chaturvedi S, Cockroft KM, Gutierrez J, Lombardi-Hill D, et al. 2021 Guideline for the Prevention of Stroke in Patients With Stroke and Transient Ischemic Attack: A Guideline From the American Heart Association/American Stroke Association. Stroke. 2021;52(7):e364-e467.

79. Ringleb PA, Bousser MG, Ford G, Bath P, Brainin M, Caso V, et al. Guidelines for Management of Ischaemic Stroke and Transient Ischaemic Attack 2008. Cerebrovasc Dis. 2008;25(5):457-507.

80. Liu L, Chen W, Zhou H, Duan W, Li S, Huo X, et al. Chinese Stroke Association guidelines for clinical management of cerebrovascular disorders: executive summary and 2019 update of clinical management of ischaemic cerebrovascular diseases. Stroke Vasc Neurol. 2020;5(2):159-76.

81. Klijn CJ, Paciaroni M, Berge E, Korompoki E, Kõrv J, Lal A, et al. Antithrombotic treatment for secondary prevention of stroke and other thromboembolic events in patients with stroke or transient ischemic attack and non-valvular atrial fibrillation: A European Stroke Organisation guideline. European Stroke Journal. 2019;4(3):198-223.
